# Endogenous Receptor Agonists: Resolving Inflammation

**DOI:** 10.1100/tsw.2007.188

**Published:** 2007-09-01

**Authors:** Gerhard Bannenberg, Makoto Arita, Charles N. Serhan

**Affiliations:** ^1^ Department of Plant Molecular Genetics, Centro Nacional de Biotecnología,CSIC, Madrid, Spain; ^2^ PRESTO, Japan Science and Technology Agency, Department of Health Chemistry, Graduate School of Pharmaceutical Sciences, University of Tokyo, Tokyo, Japan; ^3^ Center for Experimental Therapeutics and Reperfusion Injury, Department of Anesthesiology, Perioperative and Pain Medicine, Brigham and Women's Hospital, Harvard Medical School and Harvard School of Dental Medicine, Boston, MA, USA

**Keywords:** resolution, inflammation, neutrophil, arachidonic acid (AA), eicosapentaenoic acid (EPA), docosahexaenoic acid (DHA), omega-3 polyunsaturated fatty acids (ω-3 PUFA), resolvins, protectins, docosatriene, lipoxin (LX), prostaglandin (PG), human leukocytes, lipid mediators, apoptosis, chemokines, anti-inflammatory, aspirin

## Abstract

Controlled resolution or the physiologic resolution of a well-orchestrated inflammatory response at the tissue level is essential to return to homeostasis. A comprehensive understanding of the cellular and molecular events that control the termination of acute inflammation is needed in molecular terms given the widely held view that aberrant inflammation underlies many common diseases. This review focuses on recent advances in the understanding of the role of arachidonic acid and ω-3 polyunsaturated fatty acids (PUFA)–derived lipid mediators in regulating the resolution of inflammation. Using a functional lipidomic approach employing LC-MS-MS–based informatics, recent studies, reviewed herein, uncovered new families of local-acting chemical mediators actively biosynthesized during the resolution phase from the essential fatty acids eicosapentaenoic acid (EPA) and docosahexaenoic acid (DHA). These new families of local chemical mediators are generated endogenously in exudates collected during the resolution phase, and were coined resolvins and protectins because specific members of these novel chemical families control both the duration and magnitude of inflammation in animal models of complex diseases. Recent advances on the biosynthesis, receptors, and actions of these novel anti-inflammatory and proresolving lipid mediators are reviewed with the aim to bring to attention the important role of specific lipid mediators as endogenous agonists in inflammation resolution.

## INTRODUCTION

### Inflammation

The correct function of body tissues is indispensable for the proper function of the body as a whole. When tissues are injured through physical damage or are infected by exogenous microbial organisms, local and systemic responses are activated with the singular objectives to eliminate the offending factors as rapidly as possible, restore tissue integrity, and retain information about the offending agent in order to facilitate recognition and elimination on a future encounter. The outcome of these responses is a rapid physiological reaction of the body towards damage and infection, that is, inflammation. The inflammatory response effectively operates towards the removal of dead tissue and microbes, yet temporarily allows the body to deviate from homeostasis in order to recover normal functioning. Five cardinal signs of inflammation are often denoted to describe this deviation from apparent health at an ultrastructural level: *calor* (heat), *rubor* (redness), *tumor* (swelling), *dolor* (pain), and *functio laesio* (loss of tissue function)[1]. These signs are reflections of hyperemia (dilation of postcapillary venules causing increased tissue perfusion and decreased blood flow associated with tissue redness and hotness), an increase in capillary permeability (causing local fluid accumulation by exudation of serum and consequent rise in oncotic pressure), and peripheral nervous tissue stimulation by tissue swelling and increased sensitivity and perception of pain stimuli (causing increased awareness of tissue disturbance). Depending on the extent of the inflammatory response, a temporary diminution of normal organ function will result.

The inflammatory response is composed of a large number of molecular, cellular, and physiological changes that allow for the effective delivery of blood-borne leukocytes and lymphocytes to the damaged or infected tissue. Together with the local synthesis of regulatory mediators, and exudation of serum factors and acute phase proteins, a seemingly complex and multifactor, yet temporally highly scheduled, series of events takes place with the purpose to remove microbes and damaged tissue by phagocytosis, the central effector mechanism of inflammation[2]. The precise composition of components that are employed, as well as the temporal execution of cellular events during the inflammatory response by each tissue, depends on the molecular nature of microbial ligands and exposed structural components of damaged tissue. Specific molecular epitopes that "flag" tissue damage are recognized by recognition systems, such as complement, collectins, and tissue factor. Likewise, specific microbial epitopes are detected by the innate immune system via receptors localized on tissue sentinel cells, such as dendritic cells and macrophages[3]. A binary recognition of conserved microbial molecular patterns in combination with molecular components of damaged tissue has been proposed to function as an effective means to discriminate different types of damage, in the presence or absence of infection[4]. Recognition of microbial antigens also activates specific humoral and cell-mediated immunological responses targeted to inactivate particular types of microbes, and allows the building of a pool of memory lymphocytes that permits future recognition of the infectious or tissue damaging agents.

In a typical acute inflammatory response, a remarkably conserved temporal order of events appears to operate[1] (Fig. 1). After an initial increase in protein exudation, polymorphonuclear leukocytes accumulate in the inflamed tissue[5,6]. These normally circulating cells migrate to the inflamed tissue in a series of steps[7]. After first adhering to endothelial cells that line the capillaries perfusing the affected tissue, neutrophils migrate via transcellular diapedesis across the endothelium into the infected or damaged tissue[8]. Both positive cues and negative counter-regulatory signals at all steps involved in cellular migration from blood to tissue determine the magnitude of cellular infiltration and activation[9,10,11,12]. These activated neutrophils rapidly clear microorganisms and dead tissue by phagocytosis[13]. After terminating their phagocytotic function, the neutrophils die via apoptosis. Following the initial accumulation of neutrophils, a delayed infiltration of monocytes follows[1,14]. Monocytes differentiate into macrophages and on their turn remove remaining apoptotic polymorphonuclear leukocytes by nonphlogistic phagocytosis[15,16]. In this type of phagocytosis, immunomodulatory and tissue-protective mediators are released, which stop further events that maintain the evolution of inflammation. Monocyte-derived macrophages also play an important role in regulating

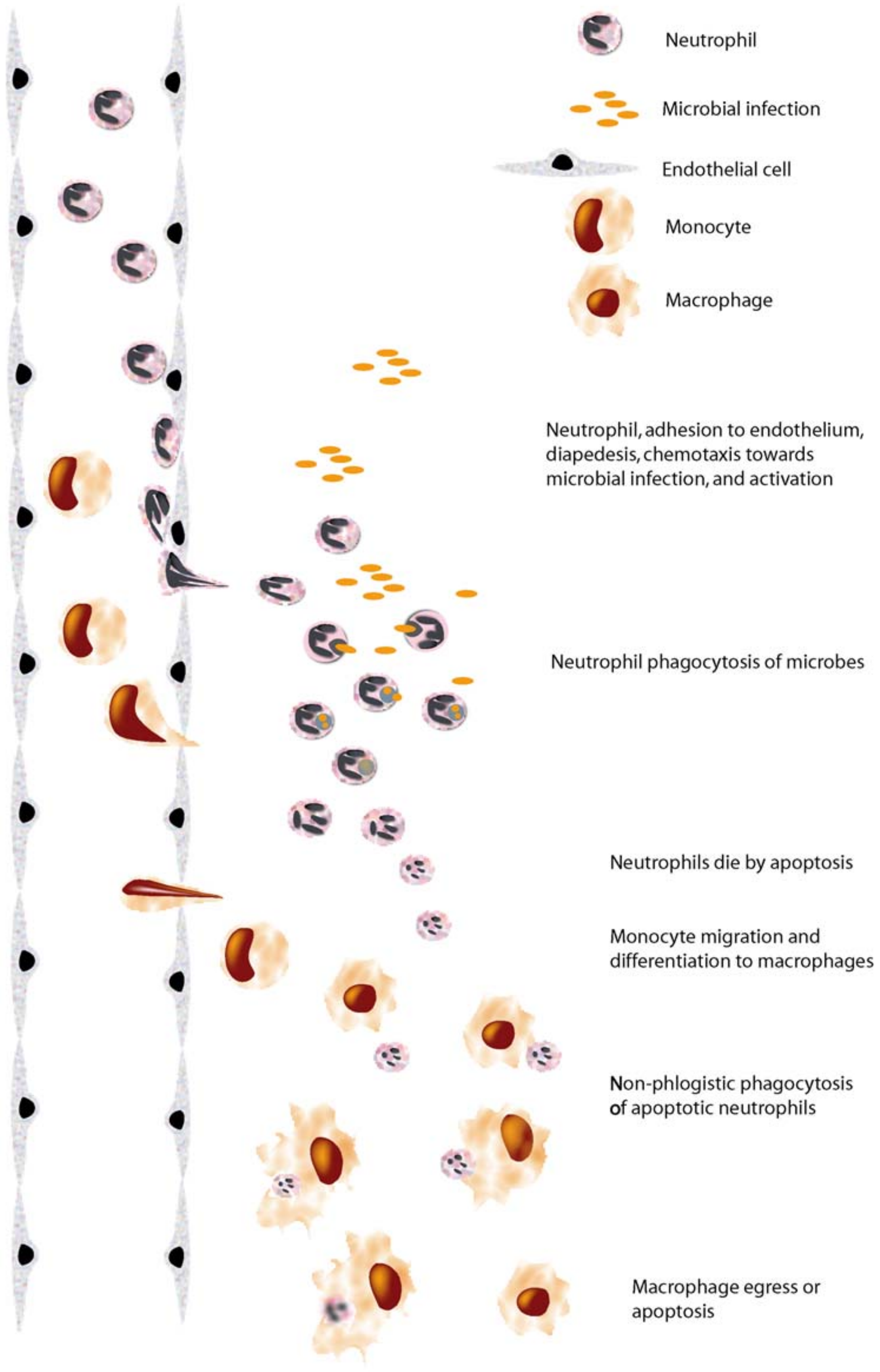

**FIGURE 1**. Representation of the temporal cellular events in the evolution of inflammation.

the subsequent restoration of tissue. Specific inflammatory cells may be called into action in particular types of infection; for example, eosinophils are particularly effective in eliminating infections by larger pathogens, such as helminthes. Lymphocytic inflammatory responses appear to become predominant during delayed-type hypersensitivity reactions. The preserved temporal order of events suggests that inflammation evolution is the activation of conserved elements that constitute a molecular program being executed according to the local needs for clearing the original inciting stimuli.

### Resolution of Inflammation

Effective clearance of microbial infections and damaged tissue is normally followed by resolution of inflammation. This process can be defined at the cellular level as the disappearance of accumulated polymorphonuclear leukocytes, and at macroscopic level as the reconstruction of tissue architecture and restoration of tissue function[1,17]. Complete resolution of tissues after bacterial infection will occur without loss of functional or structural damage and is directly related to the efficiency of microbial clearance[18,19,20]. Several mechanisms appear to drive the disappearance of inflammatory leukocytes, and operate in parallel or alternatively in different tissues. Apoptosis of leukocytes is one important route of elimination[21,22,23]. Cells that have completed their function as phagocytes undergo programmed cell death in response to local mediators that positively and negatively regulate the rate of apoptosis[24,25,26]. As polymorphonuclear leukocytes die, they simultaneously function as cytokine sinks that actively lower the level of proinflammatory cytokines from the inflammatory locus[27,28]. The apoptotic neutrophils are subsequently phagocytosed by macrophages (efferocytosis) in a so called nonphlogistic fashion, i.e., in the absence of further generation of proinflammatory mediators and stimulating the formation of anti-inflammatory mediators, such as transforming growth factor (TGF)-β, lipoxin (LX) $A_4$, and interleukin (IL)-10[16,29,30]. Another important route of elimination of leukocytes is egress from the inflamed tissue, as shown for eosinophils in pulmonary inflammation[31]. Macrophages that have completed the elimination of apoptotic neutrophils on their turn disappear from the resolving inflammatory tissue by either apoptosis or egress via the lymphatic system[32].

Overall, an important conclusion of research into inflammation and resolution over the last 10 years is that both evolution and resolution are active components of the inflammatory response[24]. Resolution is not merely the passive dilution of inflammation, but displays the features of activation of specific molecular events that contribute to removal of inflammatory cells and restoration of tissue integrity. In view of the early activation of resolution as an integral part of the normal inflammatory response, and the execution of resolution as discrete molecular and cellular events, one can consider resolution (catabasis) as an essential characteristic of inflammation. The definition of catabasis is viewed as wider than just the decline from a state of disease, but rather the return to homeostasis, and emphasizes the cellular and molecular events involved in that return, which are likely to involve a set of conserved mechanisms[33,34]. To aid in defining the anti-inflammatory and resolution-stimulating actions of local mediators and drugs, we have provided a set of indices that define the cellular events during resolution with quantitative parameters[33]. An important conclusion that has emanated from a close observation of these measurable indices is that distinct lipid mediators that are generated at precise time points during the inflammatory response not only inhibit granulocytic inflammation, but also activate and accelerate resolution. As specific receptors for these anti-inflammatory and resolution-promoting lipid mediators have been shown to exist, it is now possible to firmly put forward the idea that specific chemical mediators function as endogenous receptor agonists stimulating the resolution of inflammation[24,35,36].

Defects in the normal development of inflammation are causal factors for and/or associated with tissue damage that underlies a large number of human diseases. Key roles for inflammation have been demonstrated in diseases as varied as Alzheimer's disease and cardiovascular disease[37], as well as cancer[38], which join the well-known inflammatory disorders, such as rheumatoid arthritis and periodontal disease[39,40,41]. Uncontrolled acute inflammatory responses, or chronic inflammation with recurrent episodes of acute inflammation, are associated with tissue damage that can cause discomfort and severely compromise normal tissue function. Inflammatory responses that are too active or do not resolve can, consequently, instead of aiding the body to fight infections or restore tissue architecture, become harmful in themselves. The underlying mechanism of loss of tissue function as a result of inflammation is often ascribed to the situation where activated leukocytes generate reactive oxygen and nitrogen species, and release proteases at rates that damage tissue more rapidly than that it can restore[42,43]. For a normal execution of the inflammatory process, mechanisms must therefore exist that limit the extent of leukocyte accumulation during inflammation and tone down the magnitude of inflammation evolution. Lipid mediators derived from polyunsaturated fatty acids (PUFA) arachidonic acid (AA), and the ω-3 PUFA eicosapentaenoic acid (EPA) and docosahexaenoic acid (DHA), appear to play a particularly important role as activators of endogenous counter-regulatory, anti-inflammatory, and proresolution mechanisms. Recent studies have revealed the first molecular details of the mechanisms whereby AA- and ω-3 PUFA–derived lipid mediators act as endogenous receptor agonists to trigger the active termination of inflammation via stimulating resolution. In the following sections, a closer look will be taken at the formation and actions of several resolution-stimulating lipid mediators.

### LIPID MEDIATORS AS AGONISTS FOR RESOLUTION

#### AA-Derived Anti-Inflammatory Lipid Mediators

An important conclusion derived from studies carried out in the 1950s and 1960s that aimed to describe the chemical structure and formation of the prostaglandins, was that AA is the common endogenous precursor for the biosynthesis of a large family of bioactive lipid mediators, the eicosanoids[44,45]. AA is a substrate for both lipoxygenases and cyclooxygenases, fatty acid oxygenases that incorporate one or two molecules of molecular oxygen into a bis-allylic moiety of PUFA[45,46]. Fatty acid oxygenation by these specialized oxygenases was shown to be a required first enzymatic step in the formation of a large family of eicosanoids in animals that includes the prostaglandins, thromboxane, and leukotrienes. The generation of these lipid mediators requires the posterior modification of the primary oxygenated fatty acids by specific eicosanoid synthases, and many lipid mediators can be synthesized by a single cell type that possesses both cyclooxygenase and terminal synthase[47]. Several of the eicosanoids, such as prostaglandin (PG) $E_2$, $PGD_2$, leukotrienes, and thromboxane, were initially studied with respect to their role in inflammation and reproduction[48,49]. For example, $PGE_2$ exerts potent phlogistic (proinflammatory) actions that include vasodilation, potentiation of the vascular permeability activated by histamine and bradykinin, sensitization of nerve terminals to pain stimuli, and the generation of fever during infections[50,51]. Leukotrienes are potent chemoattractants for neutrophils and mediators of vascular permeability[48]. A major focus of research on AA-derived lipid mediators has been, and still is, the understanding of their proinflammatory actions, as well as therapeutic manipulation of their biosynthesis. Although one of the prostaglandins, prostacyclin, was identified early as exerting physiological antagonistic actions to those of thromboxane in the regulation of platelet aggregation and vascular smooth muscle contraction[52], the potential anti-inflammatory or counter-regulatory actions of the eicosanoids have been surprisingly poorly explored or disregarded[53].

#### *Lipoxins and Aspirin-Triggered Lipoxins*

The insight that different cell types interact, communicate, and act in concert in physiological settings, such as inflammation, did lead to studies in the beginning of the 1980s with the objective to determine whether oxygenated AA products formed in one cell type could serve as substrates in a second interacting cell type[54,55]. The demonstration that a second dioxygenation of 15*S*-hydroxy-eicosatetraenoic acid (15*S*-HETE) occurs in leukocytes, led to the description of a new class of eicosanoids, the lipoxins. Both $LXA_4$ and $LXB_4$ were isolated, their structures elucidated, and synthesized chemically[56,57]. Later studies confirmed that transcellular biosynthesis of $LXA_4$ is a primary route of eicosanoid biosynthesis in interacting cell types, such as neutrophils and epithelial cells, or platelets and neutrophils (Fig.5). $LXA_4$

was subsequently identified to possess potent counter-regulatory actions in inflammation[57]. An important finding was that neutrophils can store 15*S*-HETE in an acylated form in specific phospholipids pools, and which can be rapidly released on cell stimulation and made available for further dioxygenation to form a second signal, such as $LXA_4$, to regulate the function of the neutrophil[58]. In view of several identified cellular actions of $LXA_4$ that appeared to be inhibitory rather than stimulatory, $LXA_4$ was termed to act as a "chalone", definable as a substance carried though the blood, which has an inhibitory effect on tissue, or organ that responds to its action. This terminology was further refined when it was discovered that $LXA_4$ does not indiscriminately inhibit cellular responses, but rather actively stimulates leukocytes and other cells via specific lipoxin binding sites[60]. The actions of $LXA_4$ more resemble that of a signal that actively "stops" neutrophil migration and activation in inflammation[61], and, on the other hand, activates in a non-phlogistic fashion monocyte migration[62]. $LXA_4$ is now known to exert potent anti-inflammatory actions that are mediated through the activation of a specific G-protein coupled seven-transmembrane domain-spanning lipoxin receptor[36,63]. In humans, there is one $LXA_4$ receptor named ALX/FPRL1, while in mice there are at least two receptors for $LXA_4$, ALX1/Fpr-rs1 and ALX2/Fpr-rs2[36,64,65]. $LXA_4$ can be considered the first example of an endogenous amphiphilic autacoid that activates receptor-mediated molecular pathways to counter-regulate acute inflammation. The anti-inflammatory and counter-regulatory actions of $LXA_4$ operate at multiple levels in a variety of resident and mobile cell types. These include immediate actions on the regulation of signal transduction pathways activated by proinflammatory and cell growth-stimulatory mediators[66,67,68,69,70]. $LXA_4$ furthermore regulates gene transcription and transcription factor activity[71,72,73,74]. Stimulation of neutrophils and eosinophils with $LXA_4$ activates a potent inhibition of chemotaxis, adhesion and migration of these leukocytes in response to pro-inflammatory mediators[75,76,77,78]. It also potently reduces neutrophil degranulation, and regulates reactive oxygen and nitrogen species generation by leukocytes[74,79,80]. Actions of $LXA_4$ on other leukocytes include (1) the stimulation of monocyte migration, (2) stimulation of nonphlogistic phagocytosis by macrophages[81], (3) inhibition of dendritic cell migration and IL-12 formation[82], and (4) inhibition of natural killer cell cytotoxic activity[83]. In experimental animal models of inflammation, the administration of $LXA_4$ potently (low μg/kg bodyweight) reduces neutrophil accumulation in inflamed tissues, pointing to the activation of endogenous anti-inflammatory circuits by $LXA_4$[57,84]. Moreover, transgenic mice with myeloid-selective overexpression of human ALX display a markedly reduced inflammatory infiltration of leukocytes in peritonitis, which further supports the counter-regulatory role of ALX[85]. Specific actions of $LXA_4$ have been identified on epithelial cells; in the gastrointestinal tract $LXA_4$ regulates IL-8 formation and has been found to play an important tissue protective role[72,86,87]. Moreover, $LXA_4$ acts as an endogenous receptor antagonist at the cysteinyl leukotriene receptor $CysLT_1$[88]. Importantly, $LXA_4$ modulates vascular, respiratory, and nervous system physiology[89,90,91,92]. Taken together, a number of actions of $LXA_4$ appear to function in concert to counter-regulate inflammation and modulate physiology in order to favor resolution of inflammation. $LXB_4$ shares many of the actions of $LXA_4$, but the study of its actions has not received as detailed consideration as $LXA_4$. Of interest, $LXB_4$ acts via the activation of a distinct and still unknown receptor. Specific intracellular proteins such as protein kinase C[93,94] and the nuclear receptor AhR[95] have also been shown to constitute receptors for lipoxins, but the physiological relevance of these interactions remains to be investigated in full.

A diastereoisomer of $LXA_4$, 15-*epi*-$LXA_4$, is formed when AA is oxygenated by cyclooxygenase (COX)-2 when this enzyme is acetylated by aspirin, followed by a second oxygenation catalyzed by 5-lipoxygenase. This unique biosynthetic route permits the formation of a bioactive analogue of $LXA_4$, named aspirin-triggered $LXA_4$ (ATL), which is more resistant to the normal routes of metabolic degradation of lipoxins *in vivo*. These routes are oxidation of $LXA_4$ to 15-oxo-$LXA_4$ by 15-hydroxy-prostaglandin dehydrogenase, reduction to 13,14-dehydro-$LXA_4$ by 15-oxo prostaglandin 13-reductase/$LTB_4$-12-hydroxy dehydrogenase, and ω-oxidation by specialized cytochrome $P_{450}$ enzymes[96,97]. 15-Epi-$LXA_4$ displays identical biological activities as native $LXA_4$, and possess comparable affinity at the ALX receptor[36,98]. 15-Epi-$LXA_4$ has been taken as a lead for introducing structural modifications with the purpose to incorporate structural elements that further contribute to resist metabolic degradation. These stable ATL analogs that resist metabolic inactivation have been proven to be equally efficacious ALX receptor agonists compared with their parent compound, and are often more potent anti-inflammatory compounds when administered *in vivo*[36,98]. Initial studies that employed the stable lipoxin analog 15-*R*/*S*-methyl-$LXA_4$ were key to bringing forward the concept of immunomodulation by lipoxins[99]. Administration of this compound in the mouse air pouch model not only lowered the levels of proinflammatory mediators MIP-2 and IL-1β, yet also stimulated the formation of IL-4, a cytokine with potent regulatory actions on inflammatory leukocytes, such as monocytes. The reorientation by $LXA_4$ of a cytokine-chemokine axis proved to be an essential propensity of lipoxins for regulating the acute inflammatory response[100]. The aspirin-triggered lipoxin analogs also potently activate the formation in epithelial cells of the microbicidal and opsonizing protein bactericidal-permeability inducing protein[101]. Recently, stimulation of nitric oxide formation by the aspirin-triggered lipoxins has been shown to account for at least part of the anti-inflammatory actions of aspirin[102].

Further modifications to the chemical structure of $LXA_4$ have been made with the aim to confer metabolic resistance to β-oxidation, another route of biotransformation of lipid mediators[103]. The replacement of carbon-3 in the $LXA_4$ structure with an oxygen atom has provided so-called 3-oxa lipoxin analogs that maintain efficacy as anti-inflammatory compounds after oral, topical, and systemic administration, and display enhanced pharmacokinetic properties. Selected 3-oxa-15-*epi*-$LXA_4$ analogs display increased efficacy compared to analogs that are sensitive to β-oxidation in particular models of inflammation, such as second-organ lung injury and dermal inflammation[84,104]. Interestingly, in spite of the development of metabolically stable lipoxin analogs, we have recently described that even after oral administration of nanogram amounts of native $LXA_4$, systemic anti-inflammatory activity is maintained[84]. This finding likely reflects the specific and high-affinity binding by lipoxin receptors of its native ligand, recognition of which only requires localized picomolar to low nanomolar concentration. Specific lipoxin transport mechanisms, such as the voltage- and sodium-independent $LXA_4$ transporter that has been described in leukocytes[105], may potentially aid in concentrating and transporting lipoxins to their sites of action.

Importantly, the actions of $LXA_4$ and ATL are not limited to toning down or counter-regulating inflammation evolution, but also specifically stimulate resolution at several levels. By activating non-phlogistic monocytic infiltration, lipoxins facilitate the delivery of the cells that clear dying neutrophils and regulate tissue restoration[62]. Moreover, $LXA_4$ itself stimulates macrophages in their function as phagocytes of apoptotic neutrophils[81]. An early decrease in exudates levels of a range of proinflammatory cytokines and chemokines is activated by ATL, and may be explained by the recent observation that lipoxins stimulate chemokine sequestration by apoptotic neutrophils[27]. In the murine air pouch model of inflammation, $LXA_4$ is generated when neutrophils start expressing 15-lipoxygenase at the onset of resolution. At this stage, a large proportion of neutrophils are apoptotic and now complete a $LXA_4$ biosynthetic circuit together with 5-lipoxygenase that is already present in these cells[106]. In this setting, the precise temporal generation of $LXA_4$ specifically turns on resolution of inflammation. $PGE_2$ formation during the inflammatory response is critical for activating the formation of $LXA_4$ ([106], see below). Cyclooxygenase-derived prostaglandins also initiate the generation of $LXA_4$ in acid-induced acute lung injury in mice, and this action was found to be essential for the resolution of airway inflammation[107]. Additionally, macrophages exposed to apoptotic lymphocytes start to produce $LXA_4$, which may further enhance apoptotic cell removal[29]. At the same time, this process activates the generation of TGF-β, which can down-regulate a number of proinflammatory circuits[29]. The ATL stable analog ATLa2 (16-*p*-fluorophenoxy-15-*epi*-$LXA_4$-methyl ester) augments TGF-β release prior to and during the resolution phase of zymosan A–stimulated peritonitis[33].

$LXA_4$ is known to regulate the proliferation of a number of cell types, including a potent inhibitory action on lung fibroblast proliferation in response to connective-tissue growth factor, and mesangial cell proliferation stimulated by platelet-derived growth factor, tumor necrosis factor (TNF)-α, or leukotriene (LT) $D_4$[108,109]. $LXA_4$ furthermore reduces collagen biosynthesis and fibroblast growth induced by TGF-β[110]. These actions indicate that lipoxins may counteract the fibrotic response, which is one of several outcomes of tissue remodeling during inflammation. These findings most likely point to distinct temporal roles of lipid mediators such as promoting leukocyte clearance during the neutrophil-dominated resolution phase, and regulating tissue restoration after the inflammatory response resolves.

### Prostaglandin $E_2$

$PGE_2$ is a ubiquitously formed eicosanoid in mammalian tissues. At least three different enzyme systems are known that can form $PGE_2$ from $PGH_2$. Both cytosolic $PGE_2$ synthases and microsomal $PGE_2$ synthases are known, and may couple to specific cyclooxygenase-initiated biosynthetic routes[111,112,113]. The relatively widespread synthesis of $PGE_2$ matches the number of biological functions that this autacoid displays, and along with the multitude of functions is the presence of at least four $PGE_2$ (EP) receptors, EP1–4. Although $PGE_2$ is widely viewed as a proinflammatory mediator, a substantial number of studies indicate that $PGE_2$ also plays distinct roles in down-regulating immune-inflammatory responses, promotion of tissue repair, and as an activator of resolution[114]. The lung has long been known as a "privileged" site for such beneficial actions of $PGE_2$[115]. Specific actions of $PGE_2$ in stimulating resolution of inflammation have been documented in allergic edema during infection of rats with the helminth *Angiostrongylus costaricensus*[116]. High concentrations of $PGE_2$ are formed during inflammation and specifically peak at the onset of resolution in the mouse air pouch model[106,117]. This is an essential event in establishing the complete biosynthetic pathway for $LXA_4$ in a single cell type, the apoptotic neutrophil. $PGE_2$ activates the transcription of 15-lipoxygenase in exudate neutrophils during TNF-α–induced inflammation. The generation of $LXA_4$ on its turn was shown to activate the resolution of inflammation[106]. Such a distinct role for $PGE_2$ in eicosanoid class switching to form $LXA_4$ during resolution does not appear to be involved in two other models of self-resolving inflammation. In zymosan-stimulated peritonitis in mouse, $PGE_2$ levels rise during the resolution interval, but is not accompanied with an increase in $LXA_4$[33]. The $LXA_4$ concentration in the inflammatory exudate peaks during the proinflammatory phase where it may act to counterbalance $LTB_4$-activated chemoattraction, migration, and activation of neutrophils[77]. In a second well-studied model of self-resolving inflammation, carrageenin-induced pleurisy in rat, $PGE_2$ is formed during the evolution of inflammation, but is not formed at all immediately prior to or during resolution[118]. These apparent incongruous results are perhaps providing us with the first indications of the existence of distinct types of resolution responses that are activated in particular settings of inflammation. Inflammation in the air pouch is a model for a slowly healing wound that is additionally stimulated by TNF-α, whereas zymosan-stimulated peritonitis and carrageenin-induced pleurisy are models of infection with minimal tissue damage. Resolving an inflammatory response to a wound vs. a sterile infection most likely also requires a different set of molecular actors in order to effectively return to homeostasis.

### Prostaglandin $D_2$

$PGD_2$ is an important lipid mediator that acts on smooth muscle, inhibits platelet aggregation, influences pain sensation, and regulates our sleeping behavior[119]. Both the central nervous system and peripheral organs can synthesize $PGD_2$ via the isomerization of $PGH_2$ catalyzed by lipocalin-type and hematopoietic PGD synthases. $PGD_2$ has been shown to exert an important function in limiting neutrophil infiltration in the early phases of experimentally induced colitis in rat[120]. A specific function for $PGD_2$ in inflammation resolution has been indicated by a number of studies that have shown increased expression of the hematopoietic PGD synthase during resolution of heart inflammation after endotoxin treatment of mice[121], increased $PGD_2$ formation during resolution of peritonitis in mice[33], and a reduction of neutrophilic inflammation by local administration of hematopoietic PGD synthase–expressing fibroblasts in the murine air pouch model[122]. Furthermore, inhibition of $PGD_2$ formation, by inhibition of COX-2, impairs resolution of carrageenin-stimulated pleurisy in mice[118]. The role of $PGD_2$ in inflammation has become a subject of intense study after it was found nonenzymatic breakdown products of $PGD_2$ were shown to display potent anti-inflammatory actions[49,123,124]. These products are prostaglandins possessing a cyclopentenone structure and include $PGJ_2$, Δ12-$PGJ_2$, and 15-deoxy-$\Delta^{12,14}$-$PGJ_2$. Specific proresolution actions of the cyclopentenones may be of particular importance in T-helper 1 (Th1)–type inflammatory reactions, where $PGD_2$-derived compounds regulate the intensity of delayed-type hypersensitivity responses and act as endogenous braking signals for lymphocytes to stimulate resolution[125]. Δ12-$PGJ_2$, and 15-deoxy-$\Delta^{12,14}$-$PGJ_2$ also potently stimulate neutrophil apoptosis[126], their formation is triggered during phagocytosis of apoptotic neutrophils by macrophages[127], and regulate macrophage activation and proinflammatory gene expression[49]. The actions of the $PGD_2$-derived cyclopentenones appear to be induced independently from activation of the specific membrane receptors for $PGD_2$, $DP_1$, or $CRTH_2/DP_2$ receptor. Rather, their chemical reactivity permits covalent adduct formation with reactive sulfhydryl groups, a mechanism whereby these compounds regulate the activity of intracellular regulatory proteins. In particular, a number of redox-sensitive transcription factors, and their regulators, are now known to be regulated by cyclopentenone prostaglandins[128,129,130]. These include PPARγ, Nrf2/Keap1, NF-κB, AP-1, H-Ras, and p53. The activation of specific transcription factors subsequently triggers the transcription of genes that play regulatory roles in tissue protection and restoration during resolution[131]. Some points of controversy with regard to the cyclopentenone $PGD_2$ products continue to provide an interesting front of research. First of all, careful studies of the biosynthesis of $PGD_2$ products *in vivo* have indicated that the bioactive anti-inflammatory $PGD_2$ dehydration products do not appear to be formed *in vivo* during situations where $PGD_2$ is formed[132]. Furthermore, the use of supraphysiological concentrations of sulfhydryl-reactive products may have given experimental results that do not accurately reflect the *in vivo* situation. In contrast, mice deficient in hematopoietic $PGD_2$ synthase display defects in resolution of T-cell–dominated inflammation in a model of delayed-type hypersensitivity. Resolution can be stimulated in these mice by administration of 15-deoxy-$\Delta^{12,14}$-$PGJ_2$, indicating that $PGD_2$-derived cyclopentenone products do play an important role in terminating inflammation[125]. It remains a possibility that the reactive prostaglandin cyclopentenones are not detectable in their free form due to their fast rates of reaction with ubiquitous low molecular weight and protein sulfhydryl groups. Indeed, intracellular 15-deoxy-$\Delta^{12,14}$-$PGJ_2$ was detected in activated macrophages by monoclonal antibody specific to 15-deoxy-$\Delta^{12,14}$-$PGJ_2$[133]. Taken together, the $PGD_2$-derived cyclopentenone products remain of interest, because their high potency and specific modes of action point to valuable therapeutic targets for stimulation of resolution. Covalent modifications of regulatory proteins by reactive cyclopentenones also indicate that not all agonists for resolution obligatorily act through reversible high-affinity interactions with cell-surface G-protein coupled receptors. Interestingly, the formation of the $PGD_2$ dehydration products was originally found as an albumin-promoted reaction. The levels of both albumin and $PGD_2$ are both markedly increased in inflammatory exudates before and during resolution[33]. The conditions for extracellular $PGJ_2$ formation are therefore, at least theoretically, present, and detailed temporal studies on specific regulatory protein-$PGJ_2$ adducts may shed new light on the endogenous role of these potent $PGD_2$-derived anti-inflammatory compounds in resolution. Furthermore, the role of activation of the $PGD_2$ receptors $DP_1$ and $CRTH_2/DP_2$ has not been addressed with respect to their involvement in resolution.

### Prostaglandin $F_{2\alpha}$

The role of $PGF_{2\alpha}$ has been relatively little studied with respect to the resolution of inflammation. $PGF_{2\alpha}$ can be synthesized by separate reductive routes from either $PGH_2$ and $PGE_2$[134,135]. An 11-epi-$PGF_{2\alpha}$ epimer, with a different pharmacological profile from $PGF_{2\alpha}$, is formed by reduction from $PGD_2$. One recent study has indicated that COX-2–derived $PGF_{2\alpha}$ may contribute to resolution of pleural inflammation in mice[136]. In this study, an exacerbation of murine pleural inflammation during the resolution phase after inhibition of COX-2 by administration of a highly-selective cyclooxygenase-2 inhibitor was reversed by administration of the selective FP-receptor agonist fluprostenol. More detailed studies are required to demonstrate the precise role and contribution of $PGF_{2\alpha}$ as an endogenous agonist for stimulating resolution.

### ω-3 PUFA-Derived Anti-Inflammatory/Proresolution Lipid Mediators

Studies at the beginning of the 20th century already indicated the essential roles of ω-3 PUFA in health[137]. Many studies have since addressed the beneficial properties that underlie the protective action of EPA and DHA, the major ω-3 PUFA in mammalian organisms[138,139,140,141]. A large randomized trial carried out with >11,000 patients with cardiovascular disease (Gruppo Italiano per lo Studio della sopravvivenza nell'Infarto miocardico study) showed that patients taking almost 1 g of ω-3 PUFA per day displayed an approximately 45% reduction in sudden death[142,143]. Inspection of the details of this trial indicated that patients also were taking aspirin. Aspirin therapy was not taken into account for the beneficial outcomes of this trial. The human lipoxygenases (5-lipoxygenase [LO], 12-LO and 15-LO) can oxygenate ω-3 PUFA to several monohydroxylated products. The biological importance of these lipoxygenase-derived products was not known[144,145,146]. Cyclooxygenase can oxygenate EPA to form $PGH_3$, which can subsequently be transformed to analogs of the natural AA-derived prostaglandins. However, these prostaglandin-like compounds appear to be far less potent agonists for the specific prostaglandin receptors. DHA can also be oxygenated in a nonenzymatic fashion to isoprostane-like compounds (neuroprostanes) in the brain[147], or undergo autooxidation to racemic mixtures of monohydroxy compounds[148]. Early studies had indicated that cyclooxygenase does not oxygenate DHA[149]. In summary, several decades of research to understand the molecular basis for the essential roles of ω-3 PUFA in human health have not provided a clear molecular mechanism to explain their regulatory and immunoprotective roles.

#### *Resolvins*

Recently, we have identified novel oxygenated products derived from EPA and DHA that are generated via enzymatic pathways. Importantly, these compounds were identified first during the resolution phase of acute inflammation[150,151,152], namely, in a murine model of inflammation that spontaneously resolves, the dorsal skin air pouch[53,153]. Exudates were collected during the resolution phase and lipid mediator profiles were carefully recorded using LC-UV-MS/MS. Exudate cells from resolving air pouches that had been supplied with EPA and aspirin generated 18*R*-HEPE, 5-HEPE, and 5,12,18*R*-trihydroxy-EPE on stimulation of the cells with calcium ionophore (Fig. 2). Human endothelial cells expressing cyclooxygenase-2 incubated with aspirin and EPA formed 18*R*-HEPE. Incubation of 18*R*-HEPE with human neutrophils generated 5,12,18R-trihydroxy-EPE, as well as $LXA_5$[150]. 5,12,18*R*-Trihydroxy-EPE proved to be a very potent inhibitor of human neutrophil transmigration across endothelial cells towards chemoattractants, such as $LTB_4$ and fMLP[150]. Isolated cyclooxygenase-2 treated with aspirin oxygenated EPA to form 18*R*-HEPE as well as 15*R*-HEPE. Of interest is the finding that two other commonly used anti-inflammatory drugs, acetaminophen and indomethacin, were found to also permit the oxygenation of EPA by COX-2 to form 15*R*- and 18*R*-HEPE. In summary, these initial experiments indicated that vascular endothelial cells containing COX-2 acetylated by aspirin convert EPA to 18*R*-HEPE, which is oxygenated by 5-LO in human neutrophils to form a 5(6)-epoxide–containing intermediate that undergoes epoxide hydrolysis and rearrangement to 5,12,18*R*-triHEPE (Fig. 2). This compound was named Resolvin E1 (RvE1), a name that reflects the temporal frame in which biosynthesis occurs (resolution phase), as well as the spatial cellular organization (interaction) necessary for bringing together the required biosynthetic enzymes. The complete stereochemical assignment was established using biogenic and synthetic materials and proved to be 5*S*,12*R*,18*R*-trihydroxy-6*Z*,8*E*,10*E*,14*Z*,16*E*-

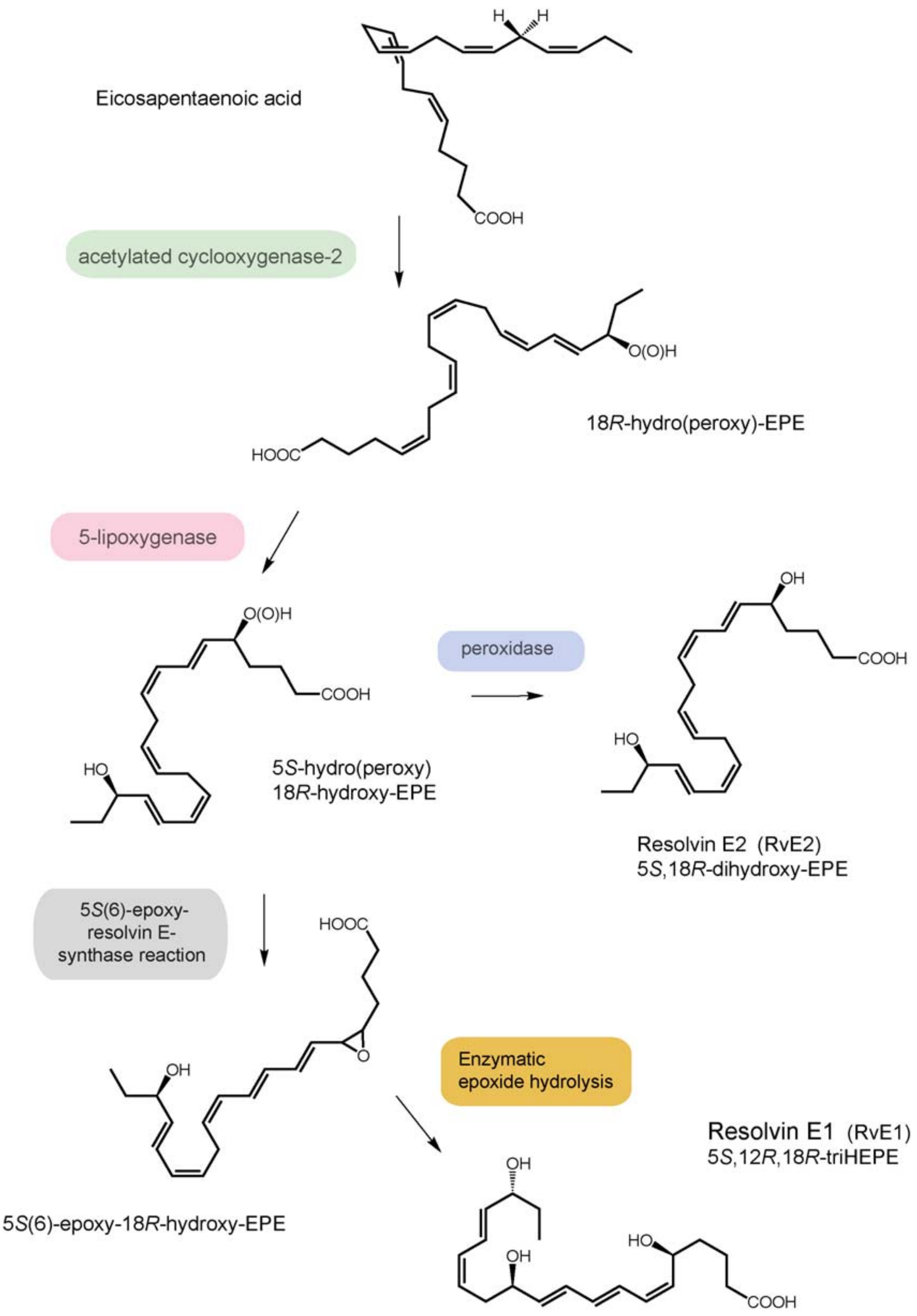

**FIGURE 2.** Biosynthesis of E-series resolvins. EPA is the endogenous substrate for the formation of E-series resolvins, RvE1 and RvE2. EPA is first oxygenated by COX-2, acetylated in its catalytic center by aspirin, to form 18*R*-hydroperoxy-EPE, which is subsequently oxygenated by neutrophil 5-LO to form 5*S*-hydroperoxy-18*R*-HEPE. RvE1 is formed via the formation of an epoxide and subsequent hydrolysis. RvE2 is formed by reduction of the hydroperoxy group.

EPA[150,154]. RvE1 displays potent anti-inflammatory actions *in vivo*; intravenous administration of 100 ng compound reduces exudate cell numbers in the murine air pouch model[33,151,154]. In zymosan A–stimulated murine peritonitis, local administration of 300 ng RvE1 reduces exudate leukocyte numbers and activates resolution at an earlier time point, without shortening the resolution interval $R_i$. RvE1 specifically reduces the exudate levels of a number of proinflammatory cytokines and chemokines (IL-6, TNF-α, KC, JE, MIP-1α, MIP-2, and RANTES) during resolution[33]. RvE1 reduces pathogen-stimulated dendritic cell IL-12 generation and migration in the spleen[154]. RvE1 also reduces *Porphyromonas gingivalis*–induced oral inflammation and alveolar bone loss during periodontitis[155], as well as has protective actions in trinitrobenzene-sulfonic acid–induced colitis in mice[156]. RvE1 binds to a G-protein coupled receptor to down-regulate the activity of NF-κB. This RvE1 receptor is expressed in myeloid, gastrointestinal, kidney, brain, and cardiovascular tissues[154]. Prior studies identified this receptor to be a receptor for the serum peptide chemerin, and is hence termed ChemR23[157]. It is also known as chemokine-like receptor-1. The inhibitory action of RvE1 on dendritic cell migration has been demonstrated to be mediated through ChemR23 activation[154]. Interestingly, human adherent monocytes display a marked ChemR23 expression, but neutrophils do not. This observation indicates that tissue monocytes/macrophages could substantially contribute to the resolution-promoting actions of RvE1. The ChemR23 receptor is up-regulated by the anti-inflammatory cytokine TGF-β[158]. Given the potent actions of RvE1 on neutrophils, the existence of a second receptor for RvE1 on human neutrophils is implied.

Just as lipoxins are susceptible to metabolic degradation to regulate tissue concentrations, RvE1 is transformed by 15-hydroxy-prostaglandin dehydrogenase in the lung to form 18-oxo-RvE1, which is inactive *in vivo*. ω-Hydroxylation of RvE1 was found to be a major route of metabolism in human neutrophils[159]. A stable analog of RvE1, 19-(*p*-fluorophenoxy)-RvE1, was synthesized to circumvent these two major routes of metabolic inactivation, and was shown to retain the anti-inflammatory properties of RvE1[159]. Recently, RvE2 (5*S*,18-dihydroxy-EPA) (Fig. 2) was identified and proved to be as potent as RvE1 to block the infiltration of neutrophils in zymosan-stimulated murine peritonitis[160].

### Asprin-Triggered D-Series Resolvins

Not only EPA proved to be a substrate for the generation of novel compounds that possess potent anti-inflammatory, immunoregulatory, and tissue-protective actions[161,162]. DHA was subsequently shown to be oxygenated by aspirin-acetylated COX-2 as well. Resolving exudates from mice given aspirin and DHA contained 17*R*-hydroxy-DHA (17*R*-HDHA)[151]. The same compound was formed by microvascular endothelial cells treated with aspirin. Recombinant COX-2 can convert DHA to 13*S*-hydroxy-DHA, but when acetylated by aspirin the oxygenation of DHA switches to forming the epimeric 17*R*-hydroperoxy-DHA. This aspirin-triggered biosynthetic pathway has been shown to operate at sites of exudate formation (murine air pouch model) and in brain. 5-LO in human neutrophils can further oxygenate 17*R*-HDHA to form 4-hydroperoxy-17*R*-HDHA and 7-hydroperoxy-17*R*-HDHA. Both of these two intermediates can undergo further transformations to the corresponding 4,17*R*- and 7,17*R*-dihydroxylated compounds via reduction of the hydroperoxy group, as well as to trihydroxylated compounds formed via an intermediate epoxy group, and subsequent spontaneous or epoxide hydrolase–catalyzed hydrolysis (Fig. 3). Potent inhibitory actions by these novel aspirin-triggered DHA-derived bioactive products (aspirin-triggered D-series resolvins) were demonstrated on IL-1β secretion by glioma cells, and on neutrophil infiltration in zymosan A–stimulated peritonitis and the murine air pouch model[151].

### Protectins and 17S D-Series Resolvins

Interestingly, neither aspirin nor exogenous DHA was required for the *in vivo* formation of D-series resolvins that are hydroxylated at the C17 position as the *S*-stereoisomers (Fig. 3)[152]. Release of endogenous DHA can be demonstrated during the inflammatory response[33], most probably via the stimulated deacylation of DHA from intracellular phospholipid pools[163]. Endogenous DHA can be converted via lipoxygenase-initiated mechanisms *in vivo* to the 17*S* alcohol-containing series of resolvins (Fig. 3) and to compounds that possess a docosatriene structure (Fig. 4)[152,161]. One specific DHA-derived lipid mediator, 10,17*S*-docosatriene, was termed protectin D1 (PD1). When it is generated in neural tissue[164], this compound is termed neuroprotectin D1 (NPD1). The complete stereochemistry

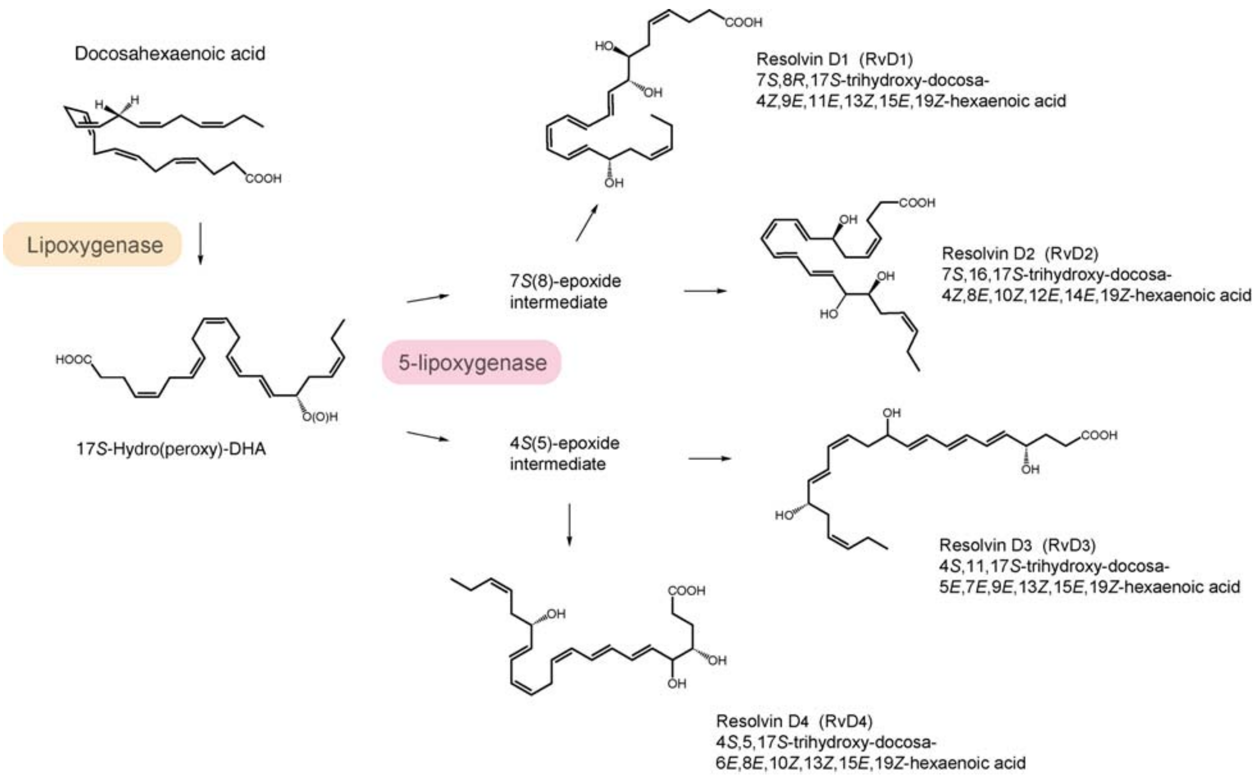

**FIGURE 3**. Biosynthesis of D-series resolvins. DHA is the endogenous substrate for the formation of novel D-series resolvins. These lipid mediators are formed by oxygenation via the action of a lipoxygenase at the carbon-17 position forming 17*S*-hydroperoxy-DHA, followed by a second oxygenation step catalyzed by neutrophil 5-LO. Note that acetylated COX-2 transforms DHA to 17*R*-hydroperoxy-DHA allowing the formation of the corresponding 17*R* D-series resolvins (aspirin-triggered resolvins).

and double bond configuration of PD1 has recently been established. This docosatriene-containing structure is formed from 17*S*-hydroperoxy-DHA via an intermediate epoxide that opens via hydrolysis and subsequent rearrangement to form (*in vivo*) 10*R*,17*S*-dihydroxy-docosa-4*Z*,7*Z*,11*E*,13*E*,15*Z*,19*Z*-hexaenoic acid[164]. PD1 formation has been demonstrated in brain, blood, Th2-skewed lymphocytes, after ischemia/reperfusion in kidney, and during the resolution phase of zymosan A–stimulated inflammation[33,152,161,165]. Potent tissue-protective and anti-inflammatory actions are exerted by PD1. PD1 reduces neutrophil accumulation in murine peritonitis after intravenous administration of 100 ng compound[152]. Neutrophil transmigration across endothelial cells was reduced approximately 50% by 10 n*M* PD1, whereas the Δ15-trans isomer of PD1 was inactive. Potent protective actions afforded by this lipid mediator have been determined in experimental models of stroke[161], and in the protection of retinal epithelial cells from oxidative stress[161,162]. Furthermore, a markedly reduced formation of PD1 and decreased 15-LO expression has been found in Alzheimer's disease brain tissue[41]. Both 17S-hydroxy-DHA and NPD1 have been shown to potently reduce TNF-α-stimulated IL-1β gene transcription in human glial cells[152].

The potent and stereospecific actions of PD1 strongly suggest that PD1 acts through a yet unidentified receptor. A further indication to the existence of a specific PD1 receptor is the observation that the inhibitory actions of PD1 are additive with those of RvE1 *in vivo*[164]. An explicit role for PD1 in resolution of inflammation has been exposed[33]; the formation of PD1 is activated specifically during the resolution phase of murine peritonitis. Local administration of 300 ng PD1 prior to initiation of zymosan A–stimulated peritonitis reduced maximal neutrophil infiltration (decreased $\psi_{max}$), shortened the resolution interval (decreased $R_i$), and activated resolution at an earlier time point (decreased $T_{max}$). Furthermore, like RvE1, PD1 reduced proinflammatory cytokine levels specifically during the resolution phase in this model of inflammation[33].

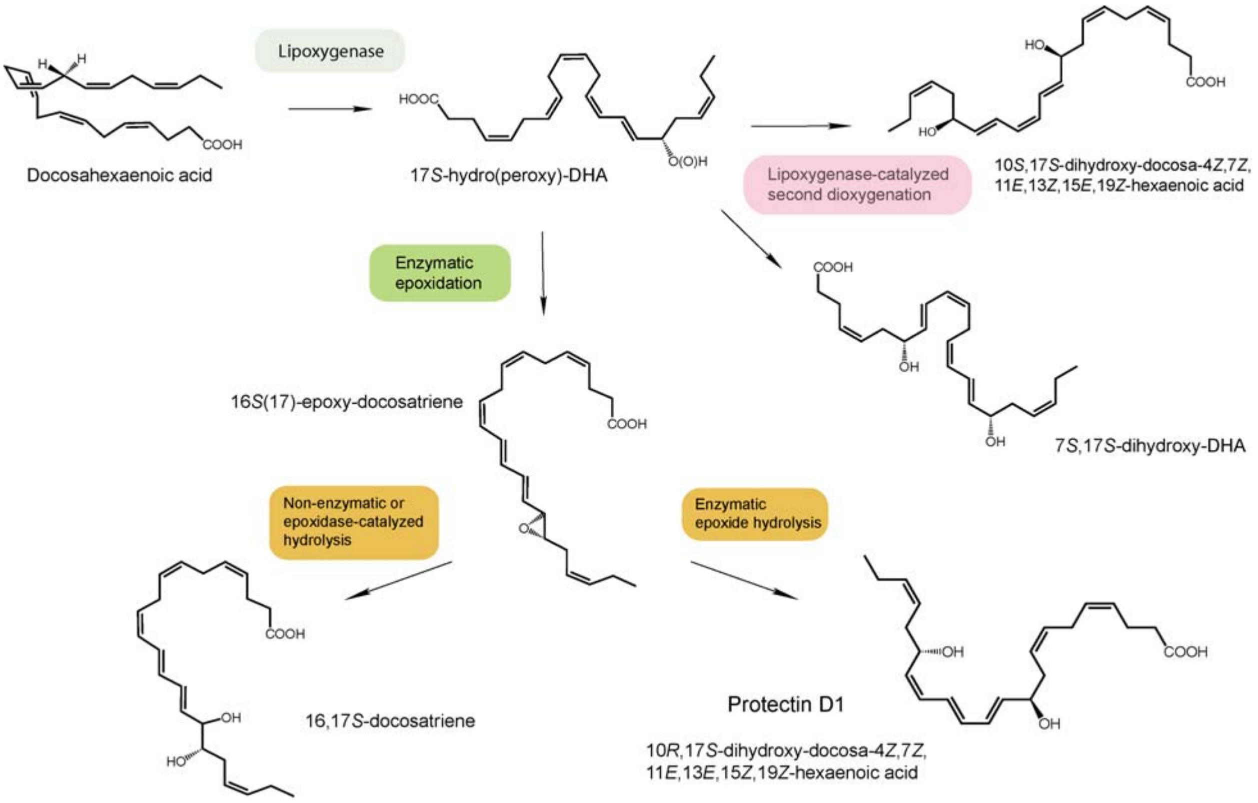

**FIGURE 4**. Biosynthesis of PD1. 17*S*-Hydroperoxy-DHA, generated by oxygenation of DHA, can also be transformed to a 16*S*(17)-epoxy-docosatriene intermediate that, via hydrolysis, leads to the formation of a 10*R*,17*S*-dihydroxy-triene–containing lipid mediator named PD1, as well as a 16,17*S*-docosatriene. A second lipoxygenation-catalyzed dioxygenation of 17*S*-hydroperoxy-DHA leads to the formation of the double-dioxygenated 10*S*,17*S*-dihydroxy geometric isomer of PD1, as well as 7,17*S*-dihydroxy-DHA. Note that aspirin-acetylated COX-2 can oxygenate DHA to form 17*R*-hydroperoxy-DHA allowing the generation of the corresponding 17*R*-epimeric aspirin-triggered products.

D-series resolvins with protective actions are also formed during inflammation. In response to bilateral ischemia/reperfusion of mouse kidney injury, small amounts of 17*S*-DHA and RvD2 (7*S*,16,17*S*-trihydroxy-DHA) were found in kidney tissue and in plasma[166]. On intravenous and subcutaneous administration of DHA, the formation of these products was markedly increased, and the formation of RvD1 (7*S*,8,17*S*-trihydroxy-DHA) and lower levels of RvD3 (Fig. 3), RvD5, and RvD6[167] were activated as well. Administration of RvD1, RvD2, and RvD3 prior to and during ischemia afforded kidney tissue protection, both functionally and morphologically; exerted a marked anti-inflammatory action; and reduced the subsequent development of interstitial fibrosis[166]. The generation of RvD1 in brain has been demonstrated after an ischemic insult to murine brain[161].

Taken together, a number of recent studies have now revealed that both EPA and DHA are endogenous precursors for the generation of a new and large family of oxygenated products that possess potent protective anti-inflammatory and resolution-stimulating actions. These actions are mediated via the activation of specific receptors, of which ChemR23 is the first identified receptor. Further support for the role of EPA and DHA as endogenous precursors for the synthesis of protective resolvins and protectins has recently been demonstrated employing transgenic Fat-1 mice that are able to synthesize ω-3 PUFA endogenously. These mice form resolvins and protectins endogenously and display a reduced inflammatory response in colitis compared to wild-type mice that are essentially dependent on dietary sources of EPA and DHA[168,169]. It is important to stress that lipid mediators of the resolvins and protectin family exert their action at picomolar to nanomolar concentrations[150,151,152,161,162], whereas EPA- and DHA-derived prostaglandin-like products, which have been characterized in earlier studies, are far less potent or devoid of biological action[144,149,170]. It is of interest that microbial organisms also possess the capacity to form oxygenated fatty acids that could feed into endogenous biosynthetic circuits forming resolvins. For instance, EPA can be converted by microbial cytochrome $P_{450}$ to form 18*R*-HEPE, which can subsequently be transformed to RvE1 by neutrophil 5-LO. Similarly, $LTB_5$ can be transformed *in vitro* to RvE1 by this cytochrome $P_{450}$ [150,171]. This route of biosynthesis was useful in the biogenic synthesis of RvE1. Whether this route is used *in vivo* in certain biological settings remains of interest. Symbiotic and pathogenic microbes have developed biosynthetic routes in coevolution with their natural hosts, and have learned to exploit the formation of anti-inflammatory, host-protective, and immunomodulatory biosynthetic routes for their survival[171,172,173].

### SUMMARY AND CONCLUSION

A physiologically normal inflammatory response can be viewed as having defined temporal phases of inflammation evolution and resolution. A number of studies in the field have now led to the idea that the inflammatory response comprises a number of programmed events that are executed in a temporally defined fashion. Several important checkpoints in inflammation can be assigned that activate the next stage of the response. It is important to note that different components may activate the employment of subsets of molecular components, or possibly in a different temporal order, depending on the original molecular stimulus that initiated inflammation. For example, we have seen that in acute inflammation initiated by administration of TNF-α in the mouse air pouch, resolution on inflammation turns on when apoptotic neutrophils start expressing 15-LO, which in concert with already present 5-LO completes the formation of a $LXA_4$ biosynthetic circuit[106]. In zymosan A–initiated murine peritonitis, $LXA_4$ appears to play a role during the evolution of inflammation, possible as a counteracting signal to regulate the magnitude of neutrophil infiltration[33]. Yet, in this model, the proresolving docosatriene PD1 is specifically formed during resolution. Together with the resolution-promoting and accelerating actions of resolvins and protectins, these findings point to central roles for endogenous lipid mediators in the activation of resolution. Delineating the different "programs" of inflammation is an important and interesting aim for future investigations, as therapeutic intervention in inflammation may need to beget precision in order to target specific components of the inflammatory response.

Inflammation underlies and accompanies a majority of the diseases that afflict human beings. Yet, treatment of inflammatory disease is generally limited to the use of compounds that antagonize the evolution of inflammation. It still remains a major challenge to treat chronic or recurrent inflammatory diseases. It is only recently that we have started to consider that the stimulation of inflammation resolution may constitute a viable therapeutic approach for treatment of inflammatory disease. We have learned that in experimental models of inflammation, resolution of inflammation is not merely the termination of inflammation, but displays discrete molecular and cellular changes that point to the active progression of inflammation to the healthy state. If the view is taken that resolution is the execution of a program that forms an essential part of the acute inflammatory response, than it follows that the temporally defined generation of pro- and anti-inflammatory and proresolution lipid mediators is an integral part of this program. Our growing appreciation that these mediators exert their biological actions

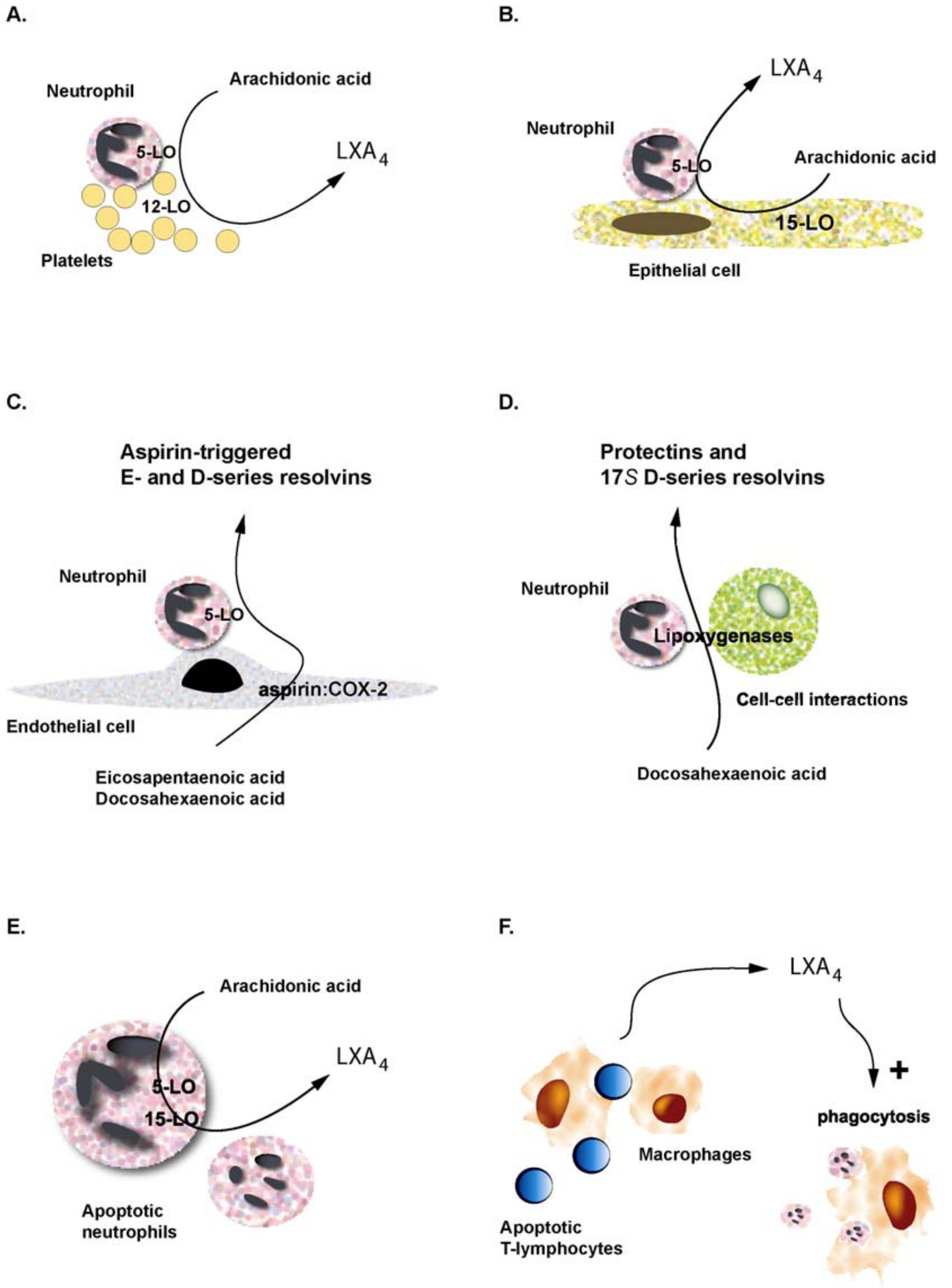

**FIGURE 5.** Generation and actions of anti-inflammatory/proresolution lipid mediators during acute inflammation and resolution. The formation of anti-inflammatory and pro-resolution lipid mediators is characterized by heterotypic cellular interactions that bring together the required fatty acid oxygenases permitting transcellular biosynthesis. (A) Biosynthesis of $LXA_4$ by transformation of AA via oxygenation by neutrophil 5-LO and platelet 12-LO. (B) Biosynthesis of $LXA_4$ by transformation of AA via sequential oxygenation by epithelial cell 15-LO and neutrophil 5-LO. (C) Formation of aspirin-triggered E- and D-series resolvins from the ω-3 PUFA EPA and DHA. (D) Formation of protectins and D-series resolvins from DHA via sequential oxygenation steps involving neutrophil lipoxygenases and other inflammatory cells lipoxygenases. (E) Formation of $LXA_4$ by apoptotic neutrophils expressing both 5- and 15-LO[106]. (F) Formation of $LXA_4$ by macrophages that are exposed to apoptotic lymphocytes[29]. The formed $LXA_4$ contributes to the nonphlogistic nature of apoptotic cell removal by phagocytosis, and $LXA_4$ itself can stimulate the phagocytosis of apoptotic neutrophils[81].

via the activation of specific receptors indicates that agonist-receptor interactions are critical regulatory events in the progression of the normal inflammatory response. Possibly, defects in the generation of the discussed proresolution lipid mediators, their agonist-receptor interactions, and subsequent signaling actions, underlie inflammatory disease. This puts forward the very interesting prospect for active modulation of life-threatening acute inflammation or chronic inflammatory disease through activation of specific endogenous resolution circuits.

### ACKNOWLEDGMENTS

Work reviewed here in the CNS laboratory was supported in part by the National Institutes of Health USA grant nos. GM38765 and P50-DE016191 (CNS). Gerard Bannenberg was supported by a Postdoctoral Fellowship from the Arthritis Foundation, and is a current Ramón y Cajal fellow supported by the Spanish Ministry of Education and Science, and the Centro Nacional de Biotecnología, Consejo Superior de Investigaciones Científicas, Madrid, Spain. Makoto Arita was supported by a Postdoctoral Fellowship for Research Abroad from the Japanese Society for the Promotion of Science and Uehara Memorial Foundation, and is a current PRESTO researcher funded by the Japanese Science and Technology Agency.
